# Local failure patterns for patients with nasopharyngeal carcinoma after intensity-modulated radiotherapy

**DOI:** 10.1186/1748-717X-9-87

**Published:** 2014-03-27

**Authors:** Jia-xin Li, Shao-min Huang, Xin-hua Jiang, Bin Ouyang, Fei Han, Shuai Liu, Bi-xiu Wen, Tai-xiang Lu

**Affiliations:** 1 Department of Radiation Oncology, Cancer Center, Sun Yat-Sen University, and State Key Laboratory of Oncology in Southern China, 651 Dongfeng Road East, Guangzhou 510060, P.R. China; 2 Department of Radiation Oncology, The First Affiliated Hospital, Sun Yat-Sen University, 58 Zhongshan Road II, Guangzhou 510080, P.R. China; 3 Imaging Diagnosis and Interventional Center, Cancer Center, Sun Yat-Sen University, and State Key Laboratory of Oncology in Southern China, 651 Dongfeng Road East, Guangzhou 510060, P.R. China

**Keywords:** Nasopharyngeal carcinoma, Neoplasm recurrence, Local, Radiometry, Radiotherapy, Intensity-modulated, Treatment failure

## Abstract

**Background:**

To investigate the clinical feature and the local failure patterns after intensity-modulated radiotherapy for nasopharyngeal carcinoma.

**Methods:**

Between March 2007 and July 2009, 710 patients with nasopharyngeal carcinoma were treated with intensity-modulated radiotherapy. The magnetic resonance imagings obtained at recurrence were registered with the original planning computed tomography for dosimetry analysis.

**Results:**

With a median follow-up of 38 months, 34 patients have developed local recurrence (32 cases valid). The incidence of invasion to nasopharynx, parapharyngeal space and the retropharyngeal space by the primary tumors was 100%, 75.0% and 62.5%, respectively, but 78.1%, 34.4% and 21.9% at recurrence, respectively. The rate of invasion to ethmoid sinus was 3.1% by the primary tumors but 28.1% at recurrence (*p* = 0.005). The topographic analysis of the local failure patterns showed "central" in 16 patients; "marginal" in 9; and "outside" in 7. The median volumes of primary gross tumor were 45.84 cm^3^ in the central failure group, 29.44 cm^3^ in the marginal failure group, and 21.52 cm^3^ in the outside failure group, respectively (*p* = 0.012), and the median volumes of primary clinical target1 were 87.28 cm^3^, 61.90 cm^3^ and 58.74 cm^3^ in the three groups, respectively (*p* = 0.033).

**Conclusions:**

In patients with nasopharyngeal carcinoma treated with intensity-modulated radiotherapy, the recurrent tumors had their unique characteristic and regularity of invasion to adjacent structures. "Central" failure was the major local failure pattern. The volumes of primary gross tumor and clinical target1 were significantly correlated with recurrent patterns. Employ more aggressive approaches to tumor cells which will be insensitive to radiotherapy may be an effective way to reduce the central failure.

## Background

Nasopharyngeal carcinoma (NPC) has a distinct epidemiology compared with other head and neck squamous cell carcinomas. Standard treatment for NPC is radiotherapy (RT) for early-stage lesions or chemoradiotherapy for more advanced diseases. However, local-regional relapse after conventional RT remains one of the major treatment failures. In researches with optimized conventional RT and a more frequent use of chemotherapy in the 1990s, the 5-year local-regional failure rate of 15.0%-19.1% has been reported [1-3]. Studies have shown that the radiation dose delivered to the target volume correlates strongly with local disease control [4]. However, dose escalation by conventional technique is limited by the tolerance of adjacent critical organs.

Intensity-modulated radiotherapy (IMRT) is a major breakthrough in the treatment of NPC. By conforming the doses to the irregularly shaped tumor, dose escalation is possible with IMRT, which can potentially lead to improvement in local control [5,6]. Early treatment outcome was encouraging and conformed the promising role of IMRT [7]. Series with longer follow-up have also demonstrated excellent outcome: the 3-year loco-regional control exceeded 90% in both early stage and advanced NPC [8,9].

However, it is critical to examine the patterns of recurrence after IMRT, since one must consider the potential that: (1) the image-based target definitions used in IMRT might lead to smaller target volumes; (2) the conformal treatment plans and radiation delivery techniques that are used to minimize the dose to adjacent normal tissues might potentially increase the risk of marginal miss. The present study investigates the regularity of recurrent tumor invading to adjacent structures and make a detailed analysis to the dosimetric relationship between the patterns of failure and the initial irradiated dose of the recurrent site, which may provide information that is helpful in the future target volume definition and margin choices.

## Methods

### Patient selection

Between March 2007 and July 2009, 710 non-metastatic NPC patients were treated with IMRT at the Cancer Center of Sun Yat-sen University. The male/female ratio was nearly 3.2:1, and age ranged from 19 to 78 years (median, 45 years). All patients underwent disease staging according to the UICC 2009 staging system [10]. A total of 29 patients were in stage I; 92 in stage II; 390 in stage III; and 199 in stage IVa-b. T-classification was as follows: T1: 89; T2: 127; T3: 329; and T4: 165.

### Radiotherapy

Patients were immobilized in the supine treatment position by a thermoplastic head and shoulder device. The CT images were obtained and transferred to the treatment-planning system, an inverse planning system (CORVUS 3.0/3.2, Peacock plan) developed by NOMOS Corporation.

Target volumes and normal tissues were delineated out on each slice. The gross tumor volume (GTV) included the primary nasopharyngeal tumor (GTVnx) and involved lymph nodes (GTVnd) as shown by clinical, endoscopic, and radiologic findings. For patients given induction chemotherapy, the targets were based on the post-chemotherapy extent as shown on the MRI images. Two clinical target volumes (CTVs) were delineated. CTV1 was defined as the GTVnx plus a 5-10 mm margin to encompass the high-risk sites of microscopic extension, including the entire nasopharynx mucosa plus a 5 mm submucosal volume. CTV2 was defined as the CTV1 plus a 5-10 mm margin (3–5 mm margin posteriorly) to encompass the low-risk sites of microscopic extension (including the parapharyngeal spaces, posterior third of nasal cavities and maxillary sinuses, pterygoid processes, pterygoid fossae, base of skull, lower half of sphenoid sinus, anterior half of clivus, and petrous apex), and lymphatic regions (the retropharyngeal lymph nodal regions, bilateral levels II, III, and Va were routinely covered in all patients, whereas ipsilateral levels IV, Vb, or supraclavicular fossae were also included for patients whose positive lymph nodes located lower than level II). Planning target volumes (PTVs) for all GTVs and CTVs were generated automatically according to the immobilization and localization uncertainties.

The prescribed dose was 68Gy to the PTVnx, 60Gy to the PTV1, 54Gy to the PTV2, and 64-66Gy to the PTVnd in 30 fractions. All patients were treated with one fraction daily over 5 days per week. The whole process of IMRT was carried out according to an institutional treatment protocol previously described [11]. The actual dose distributions for the treatment targets are listed in Table 1.

**Table 1:** Prescription dose to target volumes and dose-volume statistics

**Target**	**Goal (Gy)**	**Median below goal (%)**	**Median minimal dose (Gy)**	**Median maximal dose (Gy)**	**Median mean dose (Gy)**	**Median volume (cm**^**3**^**)**
GTVnx	68	0.18	65.57	81.24	74.82	33.62
CTV1	60	0.33	55.67	79.88	70.35	68.25
CTV2	54	2.07	39.25	78.21	63.52	322.97
GTVnd(L)	60-64	0.00	62.01	72.20	68.46	4.98
GTVnd(R)	60-64	0.00	61.95	72.15	68.02	5.96

*Abbreviations: GTVnx* primary nasopharyngeal gross tumor volume, *CTV* clinical target volume, *GTVnd(L)* GTV for the involved left cervical lymph nodes, *GTVnd(R)* GTV for the involved right cervical lymph nodes.

### Chemotherapy

Our institutional guidelines recommended IMRT alone for patients in stage I-II, and IMRT combined with concurrent chemotherapy for those in stage III-IVb. Neoadjuvant cisplatin-based chemotherapy was applied in those patients with bulky neck node (>3 cm), and adjuvant chemotherapy was used for those patients with residual disease after IMRT.

### Follow-up and statistical analysis

After IMRT completion, the patients were subsequently followed up monthly in the first 3 month, every 3 months through the first 3 years, then annually. During every follow-up visit, disease status and treatment toxicity were assessed. MRI was ordered on a regular basis and as clinically indicated. All recurrence scans were reviewed by the experienced radiologists and radiation oncologists. Patients who had complete response but developed recurrence at the primary site more than 6 months following completion of radical radiotherapy with or without chemotherapy were identified as having a recurrence. All analyses were performed in SPSS 16.0. All statistical tests were two-sided, and *p* < 0.05 was considered as statistical significance.

### Dataset registration

As has been described before [12,13], the MRI images obtained at the time when recurrence was diagnosed were input into the planning system, and each scan study became a dataset, which was a geometrically self-consistent set of image data. The initial CT dataset served as the basis for all the registrations, meaning that the MRI datasets were moved so they were registered with the initial CT geometry. Bony, vascular, and muscular structures in proximity to the failure were used to guide the co-registration process. This process was continued until satisfactory visual agreement was obtained between the MRI surfaces and CT images.

### Dosimetric and target volume analysis of the recurrence

**Figure 1:**
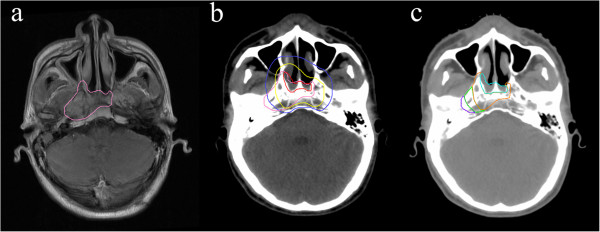
**Delineation of target volumes. (a)** The gross recurrent tumor volume (GTVr) was identified on MRI images. **(b)** The GTVr was transferred to the original planning CT. Red line is gross tumor volume (GTV); yellow line, CTV1; blue line, CTV2. **(c)** Cyan line, TG; orange line, TC1; green line, TC2; purple line, TE; TG+TC1+TC2+TE=GTVr.

The gross recurrent tumor volume (GTVr) was identified on MRI images while recurrence and transferred to the original planning CT. Further more, according to the relationship between GTVr and target volumes of the initial disease, TG (the intersection of GTVr and GTVnx), TC1 (the intersection of GTVr and CTV1, excluding TG), TC2 (the intersection of GTVr and CTV2, excluding TG and TC1) and TE (GTVr that outside the range of CTV2) were also delineated out (Figure 1), which represent recurrence in the center of the target, high-dose region, low-dose region and outside the target respectively.

**Figure 2:**
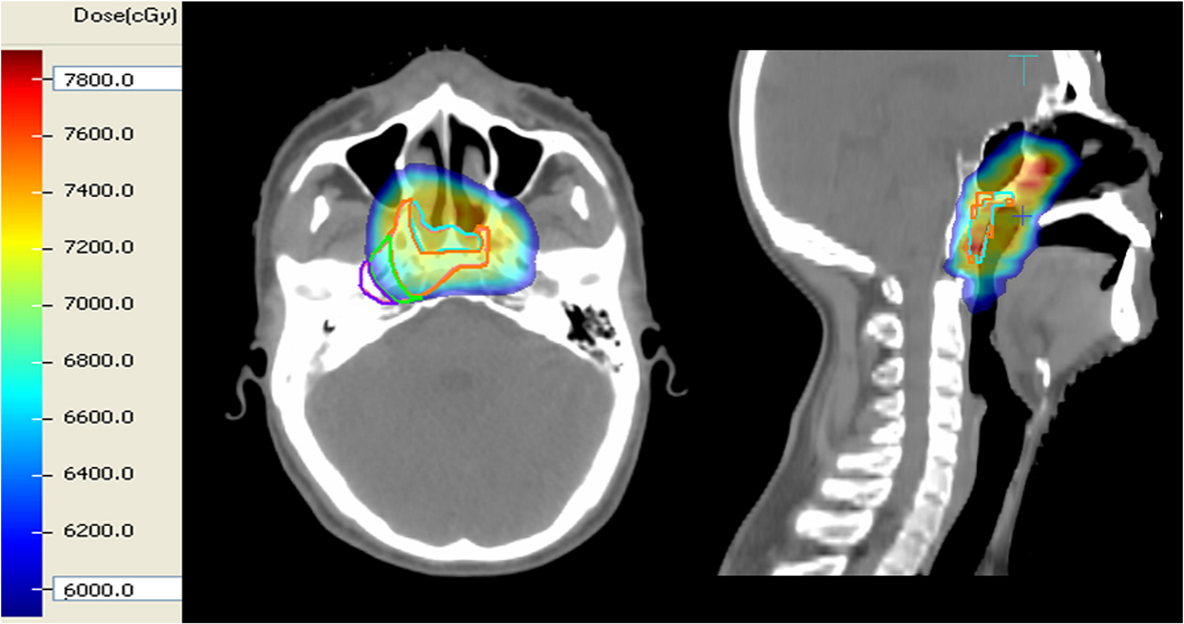
Dose delivered to the recurrent site in primary treatment.

Doses delivered to the recurrence volumes were calculated (Figure 2). Analysis of the recurrence patterns with respect to the dose distribution was initially performed by evaluating the median minimal dose, maximal dose and mean dose of GTVr, TG, TC1, TC2, TE, respectively. The percentages of the GTVr that TG, TC1, TC2 and TE accounted for were also calculated.

Three different categories were chosen for a sorting of local failure patterns:

Central: ≥50% of GTVr was within primary GTV (TG ≥50% of GTVr).

Marginal: ≥50% of GTVr was inside primary CTV but outside primary GTV (TC1 + TC2 ≥ 50% of GTVr).

Outside: ≥50% of GTVr was outside primary CTV (TE ≥50% of GTVr).

## Results

### Patient characteristics

After a median duration of 38 months (range, 6–59 months), 34 patients have developed local recurrence. In these patients, the male:female ratio was 3.6:1 and the median age was 41.5 years (range: 30–61 years). The median duration from the end of the primary treatment to the diagnosis of recurrence was 24.0 months (range: 9.0-37.0 months). Twenty-five patients had biopsy-proven recurrence, all of which were WHO III. The remaining 9 were diagnosed based on progressive symptoms associated with new image findings that would explain the symptoms, of which 4 with MRI and 5 with MRI together with [^18^F]fluorodeoxyglucose positron emission tomography scan. The usual condition was the patients with tumor over deep submucosa, basilar skull or intracranial area adjacent to the critical structures that might have technique difficulty or high morbidity probability for biopsy.

Among the 34 patients, the original IMRT plans of two patients were lost. Therefore, we concentrated our research on the remaining 32 patients. The median volume of GTVnx was 33.23 cm^3^, ranging from 6.08 to 91.43 cm^3^. The median mean doses of GTVnx, CTV1, and CTV2 were 74.82Gy, 70.50Gy and 63.52Gy, respectively.

The recurrent disease was Stage I in 4 (12.5%), Stage II in 3 (9.4%), Stage III in 11 (34.4%), Stage IVa in 12 (37.5%), Stage IVb in 1 (3.1%), and Stage IVc in 1 (3.1%) patients. A correlation between the T-stages at initial diagnosis and at recurrence (rT) was shown in Table 2. Twelve patients had the same T-stages at both diagnoses. Fifteen patients’ recurrent T-stages were more advanced than the primaries’, while 5 patients’ primary T-stages were more advanced than the recurrence’s (*p* = 0.064).

**Table 2:** Relationship between T classification at initial diagnosis and at recurrence

**Initial T classification**	**No. patients by recurrent T classification**				**Total**
	**rT1**	**rT2**	**rT3**	**rT4**	
T1	2	2	2	0	6
T2	1	1	3	2	7
T3	2	0	5	6	13
T4	0	0	2	4	6
Total	5	3	12	12	32

For further study, sites of primary and recurrent tumor invasion were compared by McNemar test (Table 3). The results showed that the invasion rates of the nasopharynx, parapharyngeal space and the retropharyngeal space by the recurrent tumor were significantly lower than those at primary. Conversely, the invasion rate of the ethmoid sinus was significantly higher at recurrence.

**Table 3:** Comparison of tumor invasion in patients with pNPC and rNPC

**Tumor invasion**	**Invasion in pNPC and rNPC (case)**	**Invasion in pNPC alone (case)**	**Invasion in rNPC alone (case)**	**No invasion in pNPC and rNPC (case)**	***P*-value**
Nasopharynx	25	7	0	0	0.008
Oropharynx	3	3	2	24	0.655
Nasal cavity	6	1	6	19	0.059
Laryngopharynx	0	0	2	30	0.157
Soft palate	0	0	3	29	0.083
Parapharyngeal space	11	13	0	8	<0.001
Longus capitis	5	9	4	14	0.052
Retropharyngeal space	6	14	1	11	0.001
Skull base	15	4	4	9	1.000
Sphenoid sinus	3	5	7	17	0.564
Ethmoid sinus	1	0	8	23	0.005
Masticator space	1	3	3	25	1.000
Cavernous sinus	2	2	3	25	0.655
Intracranial	1	0	2	29	0.157

*Abbreviations: pNPC* primary nasopharyngeal carcinoma, *rNPC* recurrent nasopharyngeal carcinoma.

As the recurrent tumor volumes were contoured within the planning system, the median mean doses of GTVr, TG, TC1, TC2 and TE were 70.86Gy, 75.00Gy, 71.27Gy, 64.39Gy and 38.10Gy, respectively, and the median mean volumes of GTVr, TG, TC1, TC2 and TE were 29.33 cm^3^, 8.74 cm^3^, 7.08 cm^3^, 2.88 cm^3^ and 0.38 cm^3^, respectively.

The topographic analysis of the local failure patterns showed "central" in 16 patients (50.0%); "marginal" in 9 patients (28.1%); and "outside" in 7 patient (21.9%).

Factors that may influence the recurrence patterns were analyzed, including the histological type at initial diagnosis, stage of initial disease, dosimetric parameters of initial IMRT plan, residue or no while initial treatment finished and interval to recurrence. It revealed only 2 significant factors: the volume of GTVnx and CTV1. The median volumes of GTVnx were 45.84 cm^3^ in the central failure group, 29.44 cm^3^ in the marginal failure group, and 21.52 cm^3^ in the outside failure group, respectively (*p* = 0.012), and the median volumes of CTV1 were 87.28 cm^3^, 61.90 cm^3^ and 58.74 cm^3^ in the three groups, respectively (*p* = 0.033).

## Discussion

IMRT is a highly conformal radiation technique enabling delivery of high radiation doses to the gross tumor and high-risk areas while sparing the adjacent organs. Since the dose distribution is significant different between IMRT and conventional RT, is there also some unique quality with the failure patterns after IMRT? This study summarized the experience through the local failed cases in large number of patients with NPC, in hopes that the application of IMRT can be further optimized.

In our patients, the majority had more advanced T-stages at recurrence than that at initial diagnosis. Nasopharynx and structures adjacent to it, such as the parapharyngeal space and retropharyngeal space, the probability of invasion could be observed to be significantly lower at recurrence. Conversely, the probability of invasion to the ethmoid sinus was significantly higher at recurrence. As the definition of CTV required, the parapharyngeal space and retropharyngeal space are usually inside the CTV1. The mean dose of CTV1 at initial radiotherapy was about 70Gy in our patients, and the biological dose would be even higher with IMRT. We speculate such a high dosage would devastate the normal structure of nasopharynx, with the local blood supply reduced and unfavorable for tumor growth, and the tumor relocates and occurs far away from the nasopharynx.

The posterior ethmoid was included in the standard two-dimensional(2-D) radiation fields [14], even patients with T1/T2 stages, so relapse in the ethmoid sinus was not common. However, IMRT can create a more conformal distribution around the targets, and the high-dose region is defined by the precise three-dimensional(3-D) sectional anatomy. So the CTV should be smaller with IMRT. Physicians initially attempted to use IMRT by creating CTV on the basis of past experiences with 2-D planning, the law of local invasion and the radiological information. There is no appreciable differences with various definitions of CTV [11,15,16], and the rates of locoregional control were similar and satisfied. But the vast majority of these definitions did not mention if the posterior ethmoid sinus should be included in the CTV.

The ethmoid sinus is the earliest developed of the nasal sinus, and it tends to spread as far as there is any compact bone, which leads to great variability with the anatomical position of the posterior border of the ethmoid sinus. It was reported that 66% of the posterior ethmoid sinus intruded into the sphenoid body, 8% intruded into the lesser wing of sphenoid bone, 38% spreaded downward and resulted in middle turbinate gasification [17]. Thus, the ethmoid sinus has a close relation with the surrounding anatomic structures. A larger sample study is expected to identify whether the posterior ethmoid sinus should be included in the high-risk area.

In the local failure patterns after 3D-RT, a study retrospectively reviewed 151 patients with head-and-neck cancer(HNC), 14 of whom developed local recurrences, which showed 12 were in-field failure, 1 was marginal, and 1 was out-of-field [18]. Since IMRT became rapidly utilized in the treatment of HNC in the late 1990s, the recurrent patterns of IMRT have been studied in limited published literature. Chao et al. analyzed 126 HNC patients treated with IMRT delivered with radical intent without surgery in 41% of patients, and post-operatively in 59% of patients [19]. After a median follow up of 26 months, 17 recurrences were noted, of which 9 were in-field, 3 were marginal failures and 5 were outside of the IMRT field. Studer et al. reported 280 HNC patients treated with IMRT, in 75% of whom definitive IMRT was performed [20]. After a mean follow-up of 23.2 months, 46 local failures have been observed, 45 of which were confirmed "in field". Ng et al. reported the treatment outcomes of 193 NPC patients with a median follow up of 30 months [21]. There were 16 local failures, 13 of that were considered in field.

In our series, local failure patterns were categorized according to the relationship of recurrent tumors and primary target volumes, which could evaluate the rationality of target volume delineation in our institution more effectively. The result demonstrated that approximately 80% of the recurrent tumors were located mainly inside CTV, with the median mean dose of GTVr as 70.86Gy. It seems that the delineation of target volumes are reasonable, but why failures in the region originally assigned to the "full dose" needs further discussion. In analysis of factors that may influence the recurrence patterns, it was found that the median volume of GTVnx and CTV1 in the group "central failure" were significantly larger than those in the other 2 groups. It is concluded that the large size tumors might not be really cured under the conventional radical dose. Shen et al. reported 154 patients with NPC treated with accelerated hyperfractionated radiotherapy [22]. The 5-year local failure-free rate were 89.4% vs 48.9% (*p* = 0.002), respectively, for patients whose GTV-P were ≤60 ml and >60 ml. In a study with 290 NPC patients [23], PTV >60 cc were associated with significantly poorer local control (*p* < 0.001). In the early 1980s, Fletcher has proposed that a certain tumor volume requires a certain radiation dose so as to get a radical cure. Tumors about 3 cm^3^ should not be irradiated less than 75Gy, and the larger ones even need more than 100Gy [24]. Therefore, larger tumors require higher doses for their control. We should design a more individual treatment plan to the large tumors, raising the prescription dose in hopes of "real cure".

A previous study showed that tumors less than 1 mm in diameter were well-oxygenated, and hypoxia would occur when the tumors grew larger [25]. Hypoxic mammalian cells are 2.5 to 3 times less radiosensitive than well-oxygenated cells. In vitro and in vivo measurements have shown that an increased radiation dose may overcome hypoxic resistance [26]. However, increasing the radiation dose indiscriminately may increase normal tissue complication rates. One approach is to implement hypoxia imaging-guided IMRT for dose escalation. So far, there were experimental application with ^18^F-FMISO,^18^F-FAZA and ^62^Cu-ATSM PET to guide IMRT planning on a small scale [27-29]. Another potential approach is to develop oxygen-mimetic, electron-affinic radiosensitizers. These radiosensitizers are easier to administer than oxygen, less rapidly metabolized, and can therefore diffuse further from blood vessels into the hypoxic regions of the tumor. Toxicity has somewhat limited their use, but there was evidence of clinical efficacy [30,31].

**Figure 3:**
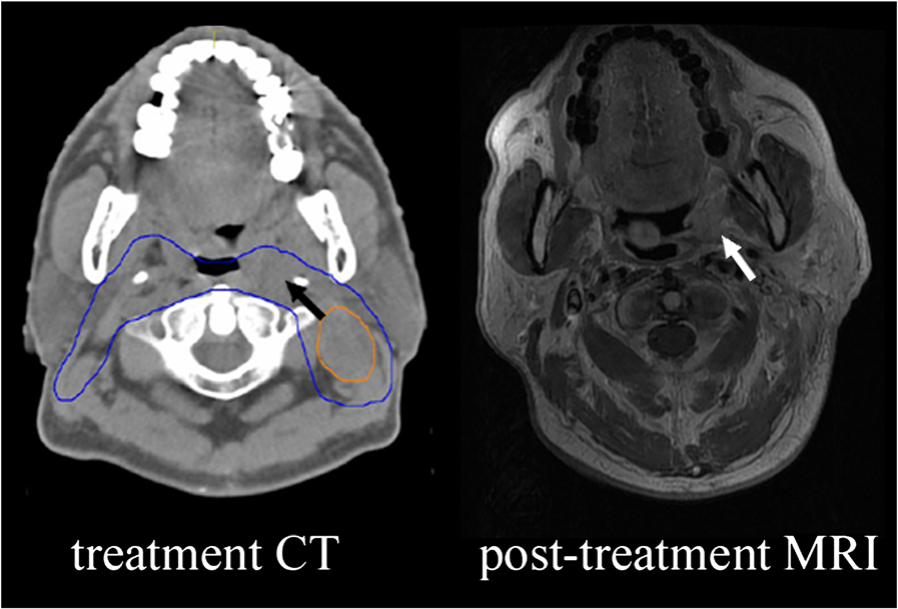
**One patient with marginal failure.** Part of primary tumor was just inside CTV2 (blue line), but outside GTV or CTV1. The white arrow indicates the recurrent tumor.

In present study, there were 9 "marginal failure" and 7 "outside the field", all of which the primary tumor extents and initial IMRT plans were reappraised. It was found that defects in target volume delineation in 4 cases. Among them, 2 primary tumors had invaded to the oropharynx and parapharyngeal space, but they were not completely included in GTV, which led to relapse in oropharynx and laryngopharynx (Figure 3). The other two patients with clivus or sphenoid bone invaded at initial diagnosis, but they were not well included in GTV, which led to recurrences in skull base and the ethmoid sinus. The high conformity and steep dose gradients with IMRT have made the accurate target volume delineation of outmost importance. Any fault or carelessness in delineation can result in overdose of the critical structure or escape of the target volume. Some measures can be adopted to improve the accuracy of target volume delineation, including establishment of guidelines for target volume delineation, obtaining high-quality image (using image fusion technology if necessary) and organizing consultations with radiologists.

There is no physiological activity like peristalsis or aspiratory movement that can cause significant anatomic changes in the nasopharynx nearby, but shrinking of the primary tumor or nodal masses, body weight loss and set-up error during fractionated RT may lead to changes in body contour, target volumes and risk organs. They would affect the dose distribution and can be the root of marginal recurrence. It was reported that patients with NPC or with great weight loss or reduction in neck separation did have clinically significant benefits with adaptive RT using helical tomotherapy [32]. We hope the benefits would eventually bring improvement in local control rate.

## Conclusions

Our study investigated the local failure patterns of NPC patients treated with IMRT in a large cohort. Based on our results, the recurrent tumors had their unique character and regularity of invasion to adjacent structures. "Central" failure was the major local failure pattern. The volumes of GTVnx and CTV1 were significantly correlated with recurrent patterns. Identify the tumor cells which will be insensitive to RT and employ more aggressive and target-specific therapeutic approaches may be an effective way to reduce the central failure.

## Consent

Written informed consent was obtained from the patient for the publication of this report and any accompanying images.

## Competing interests

The authors declare that they have no competing interests.

## Authors’ contributions

JXL carried out data collection, data analysis, manuscript drafting and revision. SMH, BO and SL contributed to the dosimetric data analysis and interpretation. XHJ aided in the image data collection. FH and BXW contributed in writing manuscript. TXL initially developed the concept of the study and contributed in writing manuscript and all revisions. All authors read and approved the final manuscript.
